# The Effect of Omega-3 Fatty Acids on Insulin Resistance

**DOI:** 10.3390/life13061322

**Published:** 2023-06-05

**Authors:** Susmita Sinha, Mainul Haque, Halyna Lugova, Santosh Kumar

**Affiliations:** 1 Department of Physiology, Khulna City Medical College and Hospital, 33 KDA Avenue, Hotel Royal Crossing, Khulna Sadar, Khulna 9100, Bangladesh; 2 The Unit of Pharmacology, Faculty of Medicine and Defence Health, Universiti Pertahanan Nasional Malaysia (National Defence University of Malaysia), Kem Perdana Sungai Besi, Kuala Lumpur 57000, Malaysia; 3 Department of Scientific Research Center (KSRC), Karnavati School of Dentistry, Karnavati University, Gandhinagar 382422, India; 4 Faculty of Medicine and Health Sciences, UCSI University Springhill (Seremban/PD) Campus, No. 2, Avenue 3, Persiaran Springhill, Port Dickson 71010, Malaysia; 5 Karnavati School of Dentistry, Karnavati University, A/907, Adalaj-Uvarsad Rd, Gandhinagar 382422, India

**Keywords:** insulin resistance, type 2 diabetes mellitus, omega-3 fatty acid, polyunsaturated fatty acid, mitochondrial dysfunction, endoplasmic reticulum stress, adipose tissue, reactive oxygen species, fish oil, inflammatory pathways

## Abstract

Insulin resistance is a critical pathophysiological process in the onset and advancement of type 2 diabetes mellitus. It is well-recognized that alterations in the metabolism of lipids and aberrant fat buildup effectively trigger the development of resistance to insulin. Adjusting one’s eating habits and managing weight appropriately are crucial for treating, controlling, and reducing the risk of T2DM because obesity and a lack of physical exercise are the primary factors responsible for the worldwide rise in T2DM. Omega-3 fatty acid is one of the polyunsaturated fatty acids (PUFA) that include long-chain omega-3 fatty acids such as eicosapentaenoic acid and docosahexaenoic acid, commonly found in fish oils. Omega-3 and omega-6 polyunsaturated fatty acids (PUFAs; 3 and 6 PUFAs) are essential for human health because they serve as metabolic precursors of eicosanoids, a class of signaling molecules that are essential for controlling a body’s inflammation. Since humans are unable to produce any of the omega-3 or omega-6 PUFAs, they both constitute imperative nutritional ingredients. Long-standing concerns about long-chain omega-3 fatty acids’ impact on diabetes management have been supported by experimental investigations that found significant increases in fasting glucose following omega-3 fatty acid supplementation and foods rich in PUFA and omega-3 fatty acid. Cellular explanations to explain the connection between inflammation and IR include mitochondrial dysfunction, endoplasmic reticulum (ER) stress, and oxidative stress. Modifications in the lipid composition of mitochondrial membranes and/or receptor-mediated signaling may be part of the mechanism behind the activation of mitochondrial fusion by fish oil/omega-3 PUFA. The exact molecular processes by which omega-3 PUFAs control mitochondrial activity to defend against IR are still unknown.

## 1. Introduction

Insulin resistance (IR) is considered the reduced responsiveness of peripheral tissues to insulin, and it was observed that IR consequences take place several years before type 2 diabetes mellitus (T2DM) [1]. T2DM raises possibilities for retinopathy, renal impairment, cardiovascular events, and amputation of the lower limb and thus becomes a primary cause of mortality [2]. Studies showed that abnormalities in insulin generation and/or function lead to a deterioration of glycemic control and culminate in the development of T2DM, which tends to result in dyslipidemia [3]. Since obesity and inadequate physical activity are the leading causes of the resultant increase in T2DM globally, adjusting one’s food-taking behavior and appropriate weight management is fundamental for curing, limiting, and reducing the risk of T2DM [4]. Hypertension, T2DM, and obesity are all factors in metabolic syndrome (MetS). The underlying causes of MetS and obesity include inflammation and disturbance of adipose tissue functioning [5]. Adipokines, bioactive peptides, and lipids secreted by adipose tissue control inflammation, insulin sensitivity, cardiovascular function, and adipose tissue function [6]. Adipose tissue secretes large amounts of interleukin-10 (IL-10) and other anti-inflammatory mediators in physically active persons [7].

On the other hand, in obese people, adipose tissue secretes a lot of pro-inflammatory adipokines, such as tumor necrosis factor (TNF alpha), monocyte chemoattractant protein 1 (MCP-1), and IL-1beta [8]. The incidence of T2DM can also be minimized if saturated fat is replaced with unsaturated fat [9]. Fatty acids serve as a source of energy, a vital component of biological membranes, and interact with various receptors and transcription factors, besides acting as precursors to paracrine mediators such as prostaglandins [10].

Glucose homeostasis is adversely affected by IR in skeletal muscle and the liver. Around 80% of postprandial glucose disposal occurs in skeletal muscle. Insulin activity and glucose homeostasis are greatly affected by the decreased glucose uptake that occurs in IR, which is predominantly caused by the inappropriate control of glucose transporter-4 (GLUT4) [11]. In the insulin-resistant state, the liver’s ability to regulate gluconeogenesis and glycogenolysis is hampered, compromising the capability to regulate glucose production. Additionally, the poor regulation of lipolysis in the white adipose tissue (WAT) is a factor in the hyperlipidemia found in insulin-resistant conditions [12].

Omega-3 fatty acid is one of the polyunsaturated fatty acids (PUFA) that include long-chain omega-3 fatty acids such as eicosapentaenoic acid and docosahexaenoic acid. These are commonly found in fish, and among the plant oils that contain alpha-linolenic acid are flaxseed, rapeseed, and canola [13]. The American Diabetes Association recommends a Mediterranean-style diet without supplementation that is rich in polyunsaturated, long-chain omega-3 fatty acids and alpha-linolenic acid [14,15]. Patients in the UK with T2DM are also encouraged to eat oily fish without supplementation [16]. PUFA should be substituted for saturated fats to reduce total and saturated fat intake and avoid diabetes [17]. Long-standing concerns about long-chain omega-3 fatty acids’ impact on diabetes management have been supported by experimental investigations that found significant increases in fasting glucose following omega-3 fatty acid supplementation and foods rich in PUFA and omega-3 fatty acid [18]. High quantities of methylmercury and polychlorinated biphenyl have been found in seafood and fish oil supplements; in addition to this, these levels impair insulin signaling and cause fasting glucose to increase in animal models [19,20]. 

Cellular explanations to explain the connection between inflammation and IR include mitochondrial dysfunction, endoplasmic reticulum (ER) stress, and oxidative stress. The mitochondria and the ER are susceptible to stress in situations involving persistent malnutrition and positive body energy balance because of excess nutrients and metabolic requirements [21]. The stress experienced by mitochondria and ER triggers the unfolded protein response (UPR) that, in turn, stimulates the main inflammatory processes and hinders insulin action [22]. Nutritional supplements and/or biologically active substances with anti-inflammatory effects may be crucial in preventing and treating insulin resistance [23]. Omega-3 polyunsaturated fatty acids (PUFA), a nutrient, have been demonstrated to have bioactive qualities associated with their recognized anti-inflammatory benefits. Investigating the molecular and cellular mechanisms of IR is a crucial area of research for the evolution of precautionary therapeutics for metabolic conditions that advance T2DM and its concurrent illnesses. Omega-3 PUFA may affect metabolic activity and the treatment of insulin resistance in individuals, but further study is required to fully comprehend how they affect inflammatory processes and cell metabolism [24].

## 2. Materials and Methods

The potential mechanisms by which dietary omega-3 PUFA alters ER stress and mitochondrial metabolic activities to stop the progression of IR are explained in this article. The electronic archiving resources used for the literature search included Google Scholar, Science Direct, PubMed, and ResearchGate. To find more literature, the references list of related works was checked. Keywords include insulin resistance, polyunsaturated fatty acids, PUFA, omega-fatty acids, omega-3 fatty acids, diabetes mellitus, cardiovascular diseases, endoplasmic reticulum stress, mitochondrial dysfunction, reactive oxygen species (ROS), oxidative stress, fish oil, randomized control trial, and inflammatory pathways. Papers authored in languages other than English and published before 2000 were excluded. The suitability of the articles was carefully assessed before they were added to the research. Duplicate articles were eliminated. Following the independent assessment and insertion of the suggested works of literature, a follow-up conversation was held to clarify any ambiguities, problems, mistakes, or biases pertaining to the specific articles.

## 3. Polyunsaturated Fatty Acid (PUFA)

**Figure 1:**
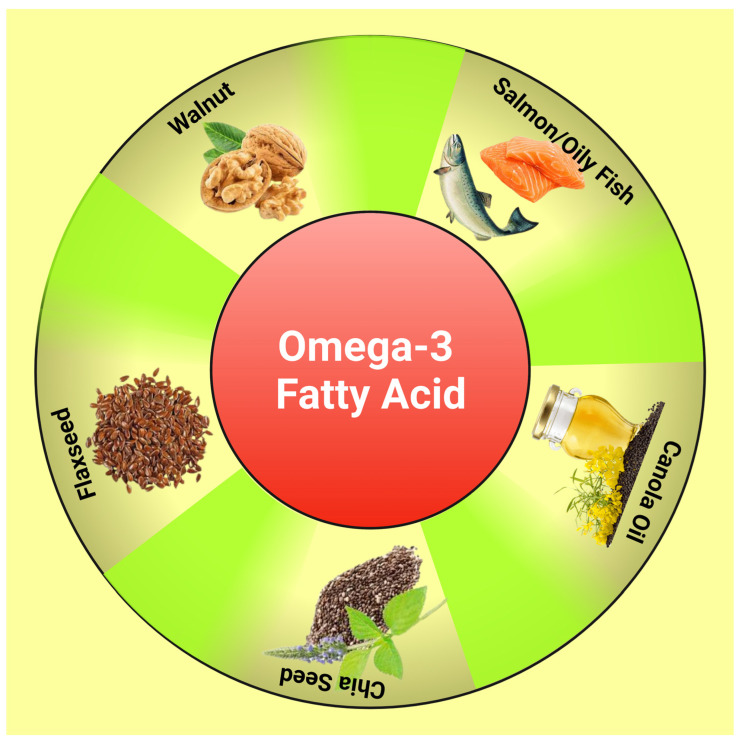
Diagram showing dietary sources of omega-3 fatty acids. This figure has been drawn utilizing the premium version of BioRender with the license number AJ25A0VPGX. Image Credit: Susmita Sinha.

PUFAs are fatty acids containing 18–24 carbons and more than 2 double bonds. A healthy body requires omega-3 and omega-6 PUFAs, which cannot be produced by the human body and should be acquired through organic foods such as walnut, flaxseed, and fish [25] (Figure 1). Many studies have revealed that omega-3 PUFAs such as eicosapentaenoic (EPA) and docosahexaenoic (DHA) can help with metabolic issues such as IR, obesity, atherosclerosis, and chronic inflammation [26]. DHA can significantly increase the number of mitochondria in muscles, lower stress before birth and oxidative damage to mitochondrial DNA, and enhance mitochondrial activity in neurodegenerative conditions [27]. The exact molecular processes by which omega-3 PUFAs control mitochondrial activity to defend against IR are still unknown.

## 4. Insulin Signaling 

**Figure 2:**
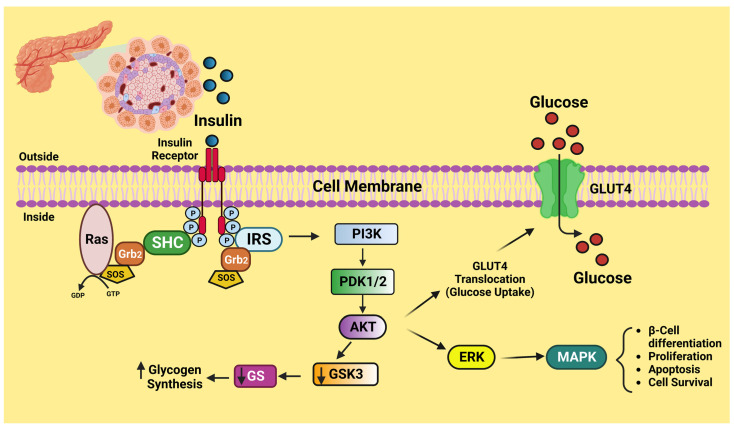
Schematic diagram of insulin signaling. This figure has been drawn utilizing the premium version of BioRender with the license number TW25A0NH90. Image Credit: Susmita Sinha.

Insulin, a peptide hormone released by the pancreatic beta-cells in response to elevated blood glucose, is the critical regulator of human carbohydrate metabolism. Insulin also prevents lipolysis in adipose tissue, which decreases the release of free fatty acids from adipose tissue (FFA) and thereby lowers blood FFA levels [28]. The insulin-sensitive tissues are muscle, adipocytes, and the liver, which undergo a cascade of intracellular signaling processes as a consequence of insulin attaching to its tetrameric receptor expressed on the cell membrane. Once insulin binds to its receptor, structural changes take place, causing some tyrosine residues to become autophosphorylated. The phosphatidylinositol 3-kinase and protein kinase B (PI3K)-AKT is triggered when the activated kinase phosphorylates tyrosine residues on insulin receptor substrates (IRS). In order to mediate insulin-induced absorption of glucose and decrease gluconeogenesis, the PI3K-AKT path is acknowledged for playing a crucial role [29] (Figure 2). The serine kinase and c-Jun N-terminal kinase (JNK) counteract the actions of PI3K-AKT. In addition, serine kinase promotes the pro-inflammatory intracellular signaling paths and functions as a negative regulator of insulin signaling [30]. When there is inflammation, the molecular signals from an inflammatory situation disrupt insulin signaling, provoking a decrease in the cell’s molecular sensitivity to insulin.

## 5. Insulin Resistance and Its Molecular Mechanism

In IR, an additional amount of insulin is necessary to facilitate the physiological impacts of insulin because the muscle cells and fat cells are unable to respond to insulin signaling sufficiently [31]. The pancreatic beta-cells enhance their insulin release to mitigate IR and restore typical blood glucose concentrations. The pancreas’ capacity to synthesize excess insulin declines gradually, and as a consequence of their exhaustion, blood glucose concentration rises [32]. An essential characteristic of the cause of T2DM is insulin resistance. Disruption of food fluxes, metabolism, and homeostasis results from the metabolically active tissues’ decreased response to insulin. At the level of molecular activity, IR is determined mainly by the abnormal collection of lipids and secondary lipid metabolites in actively metabolic tissues, notably skeletal muscle [33]. Insulin resistance progression has been linked to mitochondrial dysfunction and ER stress. The formation of ROS and the buildup of lipids as a result of mitochondrial dysfunction appeared to be significant factors causing cellular IR [34]. By modifying mitochondrial bioenergetics and ER stress, dietary omega-3 PUFA may prevent the onset of IR [35].

### 5.1. Mitochondria and Insulin Resistance

**Figure 3:**
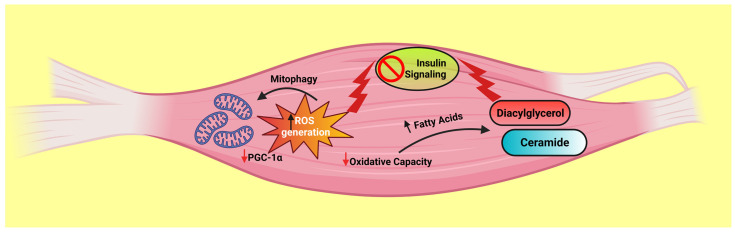
Schematic diagram showing that impaired mitochondrial oxidative capacity results in the biosynthesis of lipid metabolites such as ceramides and diacylglycerol, which have been linked to insulin resistance. This figure has been drawn utilizing the premium version of BioRender with the license number EG259YKB3J. Image Credit: Susmita Sinha.

The primary location of energy generation in mammals is the mitochondria, and malfunction of the mitochondria results in the development of a variety of illnesses, including insulin resistance, metabolic syndrome, and malignancy. Reductions in mitochondrial activity in insulin-resistant test subjects used to be the primary goal of the link connecting mitochondria and IR. Aberrant lipid buildup in peripheral organs occurs when lipid oxidation is hampered due to defective mitochondrial functioning [36]. Ceramides and diacylglycerol (DAG) are two examples of lipid metabolites that have been observed to cause stimulation of kinases that affect insulin signaling, particularly at the insulin receptor substrate-1 (IRS1) level [37] (Figure 3). The altered function of the insulin signaling pathways in skeletal muscle diminished GLUT4 exposure, which in turn reduced intracellular glucose absorption. In certain investigations, a decrease in mitochondrial mass or a decline in the ability for oxidative phosphorylation among mitochondria is related to the deterioration of the mitochondrial proficiency to oxidize [38]. However, there is still controversy over whether IR is caused mainly by mitochondrial failure.

It is hypothesized that an abundance of energy inside mitochondria, instead of increasing energy demand, leads to generating or releasing mitochondrial oxidative agents, which in turn contributes to IR [39]. Furthermore, a collection of merely oxidized acyl-carnitines, a rise in mitochondrial hydrogen peroxide (H_2_O_2_) release, and an alteration to a highly oxidizing cellular redox milieu are all caused by lipid excess within the mitochondria. This oxidized redox state could trigger IR by explicitly targeting the protein involved in the glucose uptake mechanism [40]. It is controversial to the extent that skeletal muscle mitochondrial dysfunction plays a significant part in the etiology of resistance to insulin and T2DM. However, it is widely acknowledged that this condition is characterized by a mitochondrial deficiency, which may be related to a rise in dietary lipids. Furthermore, liver and adipose tissue showed a correlation between IR and mitochondrial dysfunction.

### 5.2. Mitochondrial Dynamics and Insulin Resistance

**Figure 4:**
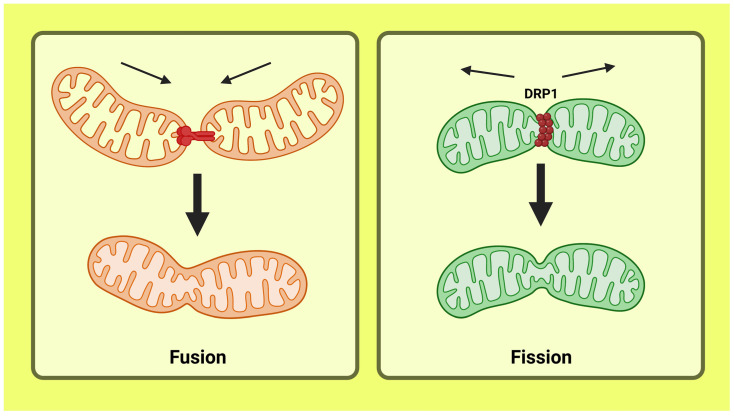
Diagram showing mitochondrial dynamics involving fusion and fission processes. This figure has been drawn utilizing the premium version of BioRender with the license number NZ259NFTT4. Image Credit: Susmita Sinha.

Mitochondria are incredibly adaptable organelles that perform various vital functions in the cell. In order to make use of substrates (such as pyruvate and NADH produced by glycolysis, acetyl co-a produced by lipid -oxidation, and glutamine) to generate energy in the form of ATP, mitochondria serve as the primary source of cellular energy generation [41]. Studies in obese and insulin-resistant people showed a reduction in skeletal muscle mitochondrial oxidative ability and faulty lipid metabolism compared to healthy, lean controls, providing the initial proof connecting mitochondrial dysfunction to resistance to insulin [42]. The multitude of extensively researched vital mitochondrial processes, including oxidative phosphorylation (OX-PHOS), calcium and reactive oxygen species (ROS) signaling, and apoptosis, emphasizes the crucial role of mitochondria [43]. Since it has been shown that they affect the performance of mitochondria, the study of mitochondrial shape and dynamics has gained considerable importance over the past ten years. The mitochondria have the capability to divide, fuse with one another, and shift across the cell. These activities are referred to as mitochondrial dynamics and entail the fission (division) and fusion processes, two different, highly regulated antagonistic activities [44] (Figure 4). Current findings suggest that mitochondrial dynamics and function are interrelated, maintaining the equilibrium between wellness and illness at the cell and organismal levels. Research is also concentrating on the crucial role of mitochondrial dynamics in the rise of resistance to insulin, precisely the harmony between fusion and fission mechanisms. The highly diverse physique of mitochondria is kept in check by an ever-changing balance within mechanisms that cause fission and fusion. These actions enable mitochondria to move locations inside a cell, exchange materials, and restore injured mitochondria [45].

The majority of mitochondrial proteins, particularly all those that entail fission and fusion, are nuclear-encoded. The mitochondrial fusion proteins mitofusins 1 and 2 (Mfn1 and Mfn2), which are positioned on the outer mitochondrial membrane [46], and the optic atrophy gene 1 (OPA1), which is situated on the inner mitochondrial membrane, both strictly control the fusion process. Dynamin-related protein 1 (DRP1), a cytosolic protein placed on the outer mitochondrial membrane by fission protein 1 (Fis1), controls the fission process [47]. It has been proposed that mitochondrial dynamic behavior is crucial for cell viability, bioenergetic function, and mitochondrial health [48]. The increased fission processes in insulin-resistant db/db mice livers were linked to mitochondrial malfunction [49]. Furthermore, high-fat-diet-fed rats exhibit lower Mfn2 gene expression than control rats, and this lower expression is coupled by diminished insulin signaling in the liver [50]. Additionally, skeletal muscle has been speculated to have a link between enhanced mitochondrial fission and fat-induced insulin resistance. T2DM and obesity were linked to lower Mfn2 expression and skeletal muscle mitochondrial size [51]. According to all these investigations, T2DM and insulin resistance are caused by alteration of mitochondrial dynamics, with the role of Mfn2 being crucial.

## 6. Endoplasmic Reticulum and Insulin Resistance

It has been proposed that the ER stress plays a significant role in the development of IR. Elevated triglyceride levels are connected to ER stress at the cell level, and this stress may induce unfolded protein response (UPR), which can lead to an inflammatory reaction. Inositol requiring kinase 1 (IRE1), activating transcription factor 6 (ATF6), and protein kinase-like ER kinase (PERK) are the three principal markers of ER stress that trigger the UPR. IRE1 phosphorylates JNK, which inhibits IRS1 (by phosphorylating it at Ser 307) and IkB kinase (IKK), which activates Nuclear factor kappa B (NFkB) and the inflammatory response, resulting in decreased insulin signaling [52]. Persistent lipid surplus causes the ER membrane to accumulate free cholesterol and phospholipids containing saturated fatty acids, which causes a modification in the composition of the membrane’s phospholipids and an aberration of fluidity. Sarcoendoplasmic reticulum Ca^2+^-ATPase 2b (SERCA2b) function is inhibited in skeletal muscle as a result of this change in the ER membrane arrangement, which induces ER Ca^2+^ deficiency and protein misfolding [53].

Although ER and mitochondria each have a unique role in the metabolic processes of cells, new research has highlighted the physiological significance of the interaction between these two organelles. The mitochondria-associated ER membrane (MAM), which connects the mitochondria and ER, ensures efficient communication between these organelles, transferring calcium ions, lipids, and other metabolites to maintain the metabolism of cells and viability [54]. 

A cytoplasmic calcium ion concentration rise in physiologic circumstances activates the insulin signaling cascade. In addition, following insulin stimulation, cytoplasmic Ca^2+^ ions in skeletal muscle and adipose tissue activate Ca^2+^/calmodulin-dependent protein kinase II (CaMKII), allowing translocation of glucose transporter 4 (Glut4) to the cell membrane [55]. Through the opening of mitochondrial permeability transition pores (mPTPs) and the triggering of inflammatory paths, the excessive retention of Ca^2+^ ions in the mitochondrial matrix induced by cellular stress promotes ROS generation, ultimately resulting in an impairment in the activity of mitochondria and apoptosis. So, it is possible to suggest that oxidative stress and/or mitochondrial dysfunction are caused by prolonged overnutrition-induced ER stress, which leads to excessive Ca^2+^ ion flow in the mitochondrial matrix and impairs cellular responsiveness to insulin [56].

## 7. The Role of Omega-3 PUFA in Insulin Resistance

Omega-3 and omega-6 polyunsaturated fatty acids (PUFAs; 3 and 6 PUFAs) are essential for human health because they serve as metabolic precursors of eicosanoids, a class of signaling molecules that are essential for controlling a body’s inflammation. The omega-3 PUFA are primarily acquired from fish but have recently been found in enhanced foods such as bread and dairy products [57]. Since humans are unable to produce any of the omega-3 or omega-6 PUFAs, they both constitute essential nutritional ingredients. Omega-3 PUFA comprises alpha-linolenic acid (ALA) and longer-chain fatty acids, eicosapentaenoic acid (EPA) and docosahexaenoic acid (DHA), which can be extracted from seafood. Because humans lack the natural desaturase enzymes necessary for its synthesis, ALA, an essential fatty acid, cannot be produced by the body and must be obtained through diet. ALA is then transformed into the long-chain omega-3 PUFA, EPA, or DHA, in the human body [58]. 

The structural components of omega-3 PUFA are necessary for forming cell walls and aid in maintaining membrane flexibility. Enhanced cell-to-cell communication and appropriate homeostasis are both supported by increasing membrane fluidity. Through its impact on cell membrane structure and/or gene expression modulation via influencing transcription factors associated with energy supply and cell cycle, omega-3 PUFA may help minimize metabolic diseases. Additionally, it has been established that the primary benefit of omega-3 PUFAs is due to their capacity to decrease inflammation, which is an essential characteristic of obesity and related metabolic diseases [59].

Numerous studies have shown that omega-3 PUFA can stop or reverse changes in the composition or function of the mitochondria in skeletal muscle. Animals fed a high-fat diet (60% fat) with fish oil (3.4% kcal from n-3 PUFAs) for 10 weeks showed increased expression of the transcriptional factors of mitochondrial biogenesis, including nuclear respiratory factor-1 (NRF1) and peroxisome proliferator-activated receptor gamma coactivator 1 alpha (PGC1a) [60]. It has been demonstrated that a diet high in fat (200 g fat/kg), including menhaden (fish) oil causes an increase in mitochondrial carnitine palmitoyl transferase 1 (CPT-1) in skeletal muscle and the hearts of rats [35]. Since CPT-I makes it easier to transport acyl groups into the mitochondria, it serves as the primary control point for beta-oxidation. The expression of CPT-I is governed by peroxisome proliferator-activated receptors (PPARs) and the 5′-AMP-activated protein kinase (AMPK) [61]. The activation of AMPK by EPA (200 mumol/L for 24 h) in primary cultured rat fat cells and the skeletal muscle of high fat-fed rats supplemented with 10% v/w omega-3 PUFA (as fish oil) for six weeks both led to a noted rise in mitochondrial CPT-1 expression and fatty acid oxidation [35]. An essential factor in preventing the establishment of insulin resistance is improved fatty acid consumption, which is likely to reduce excessive lipid buildup and lipotoxicity [62].

Higher expression of uncoupling protein 3 (UCP-3) mRNA in skeletal muscle and increased expression of peroxisomal acyl-CoA oxidase (PACO) in the liver, skeletal muscle, and heart is another explanation suggested to explain the effects of omega-3 PUFA on adipose tissue. A recent study found that feeding obese mice a high-fat diet rich in fish oil (60 percent of calories should come from fat for 12 weeks) enhanced their mice’s glucose tolerance and insulin sensitivity [63]. Reduced adipose tissue dysfunction and inhibition in high-fat-feeding-induced ER stress were linked to this increased insulin sensitivity [64]. The inhibition of ER stress by omega-3 PUFA in adipocytes has been attributed to AMPK activation [35].

Nucleotide-binding and oligomerization domain-like receptor, leucine-rich repeat, and pyrin domain-containing 3 (NLRP3) inflammasome have been proposed to be influenced by omega-3 PUFA in obesity and obesity-related metabolic disorders, such as insulin resistance [65]. Exposure to pathogens and host danger signals, inflammasomes, and multiprotein complexes mediate the immune system’s first line of defense by activating caspase-1 and inducing the release of IL-1 and IL-18 [66]. According to certain theories, NLRP3 often detects cellular homeostasis disturbances such as redox status shifts or ER stress [67]. However, the exact process by which NLRP3 becomes active is still not entirely known. ROS generation and mitochondrial damage can result from a substantial mitochondrial Ca^2+^ influx. Consequently, Ca^2+^ fluxes may be the intermediary process bridging ER stress, inflammation, and mitochondrial dysfunction paths in the progression of resistance to insulin, and omega-3 PUFA may control this process [68].

## 8. Omega Oil and Diabetes Mellitus 

The randomized control trials relating to omega oil and diabetes mellitus published within the last 5 years and indexed in PubMed are depicted in Table 1. 

## 9. Omega-3 FA and Nonalcoholic Fatty Liver Disease

Research has shown that docosahexaenoic acid (DHA) and eicosapentaenoic acid (EPA) can help nonalcoholic fatty liver disease (NAFLD) patients reduce liver fat. DHA and EPA reduce hepatic de novo lipogenesis (DNL) and distribute fatty acids away from triacylglycerol (TAG) production and toward oxidation [89]. The DNL pathway is downregulated, and oxidation is upregulated due to EPA and DHA’s effects on several hepatic transcription factors. The cumulative consequence is less hepatic TAG collection and lower circulatory TAG concentrations [90]. Fatty acid oxidation, de novo lipogenesis, lipid uptake from the bloodstream and its transformation into lipids, and very low-density lipoprotein (VLDL) assembling and release are the main processes controlling the liver’s lipid status. Omega-3 PUFA has an impact on all of these processes, at least partially, by influencing their function and/or large amounts of transcription variables that influence the expression of the genes of encrypting the proteins associated with these processes [89]. Additionally, research showed that taking supplements of omega-3 PUFA enhanced processes related to cellular respiration, ER stress, lipid metabolism, and the extracellular matrix [91]. Furthermore, a few rises in red blood cell (RBC) omega-3 PUFA content had a beneficial impact on elevated hepatic sensitivity to insulin but were negatively correlated to a decrease in fasting DNL, VLDL TG, and beta-hydroxybutyrate [92].

## 10. Conclusions

A growing number of studies have been conducted on omega-3 PUFA’s health benefits. Experiments on mice, as well as on humans, have shown that omega-3 PUFA may have impacts that assist in avoiding or alleviating obesity and related metabolic diseases. These consequences include increased fat oxidation, decreased fat accumulation, and increased energy expenditure. Modifications in the lipid composition of mitochondrial membranes and/or receptor-mediated signaling may be part of the mechanism behind the activation of mitochondrial fusion via fish oil/omega-3 PUFA. Additional research is required to comprehend this mechanism and the mechanism connecting mitochondrial fusion to improved cellular energy metabolism.

## Figures and Tables

**Table 1:** Summary of PubMed-indexed randomized clinical trials of the last 5 years.

Authors Name	Journal Details	Study Details (Study Design/Study Subjects/Dose and Duration/Effect)	Background	Result	Conclusion
Nidia et al. [69]	*J Periodontol*. 2020;91(10):1318–1327.	Placebo-controlled, double-blind, randomized clinical study/human subjects/test group (TG)1: 3 PUFA + aspirin (3 g of fish oil per day + 100 mg of aspirin per day for two months), or test group (TG)2: 3 PUFA + aspirin (3 g of fish oil per day + 100 mg of aspirin per day for two months) prior to periodontal exfoliation/both test groups’ IFN- and IL-8 levels dropped with time. For test group 1, IL-6 levels were lower, and HbA1c values dropped.	Low-dose aspirin (ASA) and omega-3 polyunsaturated fatty acid (-3 PUFA) supplements have been suggested as a host modulation strategy to manage chronic inflammatory disorders.	Clinical attachment gain for TG1 occurred in moderate and deep pockets. IFN- and interleukin (IL)-8 levels dropped with time for both test groups, and IFN- and interleukin (IL)-8 levels dropped with time.	Patients with type 2 diabetes who receive adjunctive -3 and ASA following periodontal debridement benefit clinically and immunologically from the medical management of periodontitis.
Djoussé et al. [70]	*JACC Heart Fail*. 2022;10(4):227–234.	Randomized, double-blind, placebo-controlled trial/human subjects/1 g of fish omega-3 fatty acids per day and 2000 IU of vitamin D3 (cholecalciferol) every day from 2011 to 2017/omega-3 supplementation reduced recurrent HF hospitalization exclusively in Black subjects.	It is uncertain if race and T2DM affect how often people have heart failure (HF) after taking omega-3 supplements.	The HR for the first HF hospitalization when omega-3 supplements were compared to placebo was 0.69 (95% CI: 0.50–0.95) in patients with prevalent T2D and 1.09 (95% CI: 0.88–1.34) in participants without T2D (*p* for interaction = 0.019).	Omega-3 fatty acid supplements had a beneficial impact on the frequency of HF hospitalization in individuals with T2DM but not in those without T2DM. These advantages seemed to be more pronounced in Black participants with T2DM.
ASCEND Study Collaborative Group; Bowman et al. [71]	*N Engl J Med*. 2018;379(16):1540–1550.	Randomized, double-blind, placebo-controlled trial/human subjects/1 g capsules containing either omega-3 fatty acids or a matching placebo (olive oil) daily for 4.4 years/there were no discernible variations in the frequency of major nonfatal complications comparing groups.	Increased intake of n-3 fatty acids has been associated with a reduced risk of cardiovascular disease in observational studies, but this finding has not been confirmed in randomized trials. It remains unclear whether omega-3 fatty acid supplementation has cardiovascular benefits in patients with diabetes mellitus	A major vascular incident occurred in 689 patients (8.9%) in the fatty acid group during a mean follow-up of 7.4 years (adherence rate, 76%) and in 712 patients (9.2%) in the placebo group (rate ratio, 0.97; 95% confidence interval (CI), 0.87 to 1.08; *p* = 0.55).	There was no discernible difference in the incidence of major vascular events among diabetic individuals without signs of cardiovascular disease between those who were given n-3 fatty acid supplements and those who received a placebo.
Raygan et al. [72]	*Phytother Res*. 2019;33(7):1943–1951.	Randomized control trial/human subjects/for a period of 12 weeks, 3 intervention groups were given either 1000 mg of omega-3 fatty acids from flaxseed oil, 1000 mg of omega-3 fatty acids from fish oil, or a placebo/comparable to fish oil; flaxseed oil reduced insulin while enhancing total nitrite and total antioxidant properties.	In diabetic patients with coronary heart disease, this study examined the effects of flaxseed and fish oil supplementation on cardiovascular risk factors.	Compared to the placebo, flaxseed and fish oil supplementation significantly decreased insulin levels (0.04).	This study indicated that supplementing with fish and flaxseed oils had advantageous effects on specific metabolic profiles. According to this research, flaxseed oil has a similar impact to fish oil in terms of lowering insulin and raising total nitrite and antioxidant capacity.
Dakin et al. [73]	2020;23(10):1340–1348	Randomized control trial/human subjects/individually randomized to receive 20 mg/day atorvastatin or omega-3 EE90 or a matching placebo observed over the first 16 weeks of the 1-year trial/atorvastatin would be more cost-effective than omega-3 fatty acid.	More elaborate factorial designs and the necessity for extrapolating results after the trial’s end complicates the analytical approaches. From the viewpoint of the UK National Health Service, this study evaluated the cost-effectiveness of atorvastatin, omega-3 fish oil, and an action-planning leaflet, both individually and collectively.	The results were unaffected by various methods of factorial design analysis. At GBP 20,000/QALY, there was a 99% likelihood that atorvastatin would be more cost-effective than omega-3 fatty acid.	Atorvastatin monotherapy was the most economical option when comparing the three study therapies on a GBP 20,000/QALY basis. Omega-3 fish oil was not cost-effective, and there was insufficient information to make definite statements on action planning.
Liu et al. [74]	*Am J Clin Nutr*. 2018;108(2):256–265.	Randomized controlled trial/human subjects/low-protein diet with low ω-3 PUFAs (control), a low-carbohydrat–high-protein (LCHP) diet, ω-3, or LCHP+ω-3 diet for 12 wk/greater reductions in fasting glucose levels and glycated hemoglobin (HbA1c) were seen.	It is unknown how people with type 2 diabetes (T2D) would respond to a low-carb, high-protein (LCHP) diet paired with omega-3 (n-3) polyunsaturated fatty acid (PUFA) supplementation.	Compared with the control diet group, more decreases in glycated hemoglobin (HbA1c) and fasting glucose were detected in all the other 3 diet groups at 12 weeks.	There may be a need to combine an LCHP diet with omega-3-3 PUFAs in managing T2D because the LCHP+omega-3 diet had more substantial effects on HbA1c and fasting glucose and faster effects on fasting glucose than both the LCHP and omega-3 diets.
Jamilian et al. [75]	*Br J Nutr.* 2020;123(7):792–799.	Randomized controlled trial/60 women with GDM/consumption of either 2 × 1000 mg/d omega-3 fatty acids from flaxseed oil containing 400 mg α-linolenic acid in each capsule (n = 30) or placebo (n = 30) for 6 weeks/omega-3 fatty acid supplementation for 6 weeks demonstrated positive benefits on gene expression linked to insulin, lipids, glycemic management, inflammatory markers, and oxidative stress in women with GDM.	Researchers looked at how n-3 fatty acids from flaxseed oil affected patients with genetic and metabolic gestational diabetes mellitus (GDM) characteristics.	In peripheral blood mononuclear cells from people with GDM, n-3 fatty acid intake increased PPAR- gamma (*p* < 0.001) and LDL receptor (*p* = 0.004) and decreased the gene expression of IL-1 (*p* = 0.002) and TNF-alpha (*p* = 0.001). Additionally, as compared to the placebo, n-3 fatty acid supplementation decreased fasting plasma glucose (*p* = 0.001), insulin levels (*p* = 0.001), insulin resistance (*p* = 0.001), and enhanced insulin sensitivity (*p* = 0.005).	Omega-3 fatty acid supplementation demonstrated positive benefits on gene expression linked to insulin, lipid and inflammation, glycemic control, lipids, inflammatory markers, and oxidative stress in women with GDM for six weeks.
Britten-Jones et al. [76]	*Diabetes*. 2021;70(8):1794–1806.	Randomized, double-musked, placebo-controlled trial/human subjects with type 1 DM/omega-3 (1800 mg/day fish oil) or placebo (600 mg/day olive oil) supplements for 180 days/in type 1 diabetes, long-chain omega-3 supplements promoted corneal neuro-regeneration.	Omega-3 (n-3) fatty acid oral supplementation’s effects on peripheral nerves in type 1 diabetes were examined in this placebo-controlled experiment.	Compared to the placebo, the Omega-3 Index increased by 3.3% (95% CI: 2.4, 4.2). Most functional properties of tiny and large nerve fibers were similar.	This randomized controlled research discovered that long-chain n-3 supplements cause neurodegenerative changes in the corneas of people with type 1 diabetes, suggesting a potential function in regulating the health of peripheral nerves.
O’Mahoney et al. [77]	*Cardiovasc Diabetol*. 2020;19(1):127.	Randomized controlled trial/human subjects with type 1 DM/3.3 g/day of encapsulated omega-3 PUFA or encapsulated 3.0 g/day corn oil placebo for 6 months, with follow-up at 9 months after 3 months washout/in subjects with T1D, daily high-dose bolus omega-3 PUFA supplementation did not improve glucose homeostasis, vascular health, or metabolic markers.	The significance of omega-3 polyunsaturated fatty acids and the possible effects of omega-3 PUFA supplementation in managing and treating type 1 diabetes (T1D) is still unclear and debatable.	Overall acquaintance with omega-3PUFA over the 6 months was 14.27 ± 3.05% per month under omega-3 PUFA and 9.11 ± 2.74% per month below PLA (*p* < 0.001). VCAM-1, ICAM-1, E-selectin, P-selectin, pentraxin-3, VEGF, TNFα, CIMT, FMD, blood pressure, HbA1c, FPG, and postprandial metabolism did not vary between or within groups after treatment (*p* > 0.05).	According to this study, supplementing with omega-3 PUFAs at a high dose each day for six months did not improve vasculature condition, glucose homeostasis, or metabolic parameters in Type 1 diabetes patients.
Naeini et al. [78]	*Nutr Metab Cardiovasc Dis.* 2020;30(3):441–447.	Randomized controlled trial/human subjects with T2DM/2400 mg/d DHA-rich fish oil or placebo for 8 weeks/short-term administration of docosahexaenoic acid (DHA) rich fish oil may modify PPAR activity in peripheral blood mononuclear cells.	Omega-3 polyunsaturated fatty acids (PUFAs) are peroxisome proliferator-activated receptor gamma (PPAR-γ) ligands.	Raised peroxisome proliferator-activated receptor gamma (PPAR-gamma) activity in peripheral blood mononuclear cells (PBMCs) (*p* = 0.01) compared to placebo (*p* = 0.4), while mRNA expression levels of the p53 and nuclear factor kappa-B (NFk-B) genes did not demonstrate significant variations between the study groups.	Short-term DHA-rich fish oil supplementation may modulate PPAR-γ activity in PBMCs.
Liu et al. [79]	*Lipids Health Dis*. 2022;21(1):20.	Randomized double-blinded trial/human subjects with T2DM/administration of fish oil at a dose of 3 g/day containing eicosapentaenoic acid (EPA) and docosahexaenoic acid (DHA), perilla oil at a dose of 3 g/day containing α-linolenic (ALA), linseed, and fish oil with mixed linseed and fish oil at a dose of 3 g/day containing EPA, DHA, and ALA for six months/perilla oil administration reduced fasting blood glucose levels while fish oil supplement reduced TG levels.	The composition of erythrocyte fatty acids is influenced by dietary fatty acid consumption, and abnormalities of glycolipid metabolism are highly associated with this.	There were significant effects of treatment–time interaction on fasting blood glucose (FBG), insulin, HOMA-IR and C-peptide, total cholesterol, and triglycerides (TG), and there were significant effects of time–treatment interaction (*p* = 0.001). Following the intervention, serum TG in the fish oil group dramatically dropped (*p* = 0.001), as did glucose and C-peptide in the perilla oil and linseed and fish oil groups.	Perilla oil supplementation reduced fasting blood glucose, while fish oil supplementation reduced TG. Omega-3 PUFAs from plants and animals have diverse impacts on the erythrocytes’-controlled glycolipid metabolism and fatty acid compositions.
Burhop et al. [80]	*Nutrients*. 2022;14(2):396.	Randomized controlled trial/43 obese human subjects/the calanus oil group received LC n-3 FAs-rich oil extracted from *C. finmarchicus*, while the control group was supplemented with placebo capsules (filled with paraffin oil)/in obese people, calanus oil improves insulin resistance and glucose metabolism.	*Calanus finmarchicus* oil is a source of long-chain omega-3 PUFAs that have shown encouraging results on glucose homeostasis in preclinical investigations because of its anti-obesity and/or anti-inflammatory qualities.	Fasting insulin, HOMA-IR, and the hepatic insulin resistance index significantly improved after a 12-week CO intervention. At the same time, there were no differences in HbA1c, AUC0–2 h glucose, AUC0–2 h insulin, 2 h plasma glucose, or muscle insulin sensitivity index.	In obese people, calanus oil improves insulin resistance and glucose metabolism.
Abbott et al. [81]	*Prostaglandins Leukot Essent Fatty Acids*. 2020; 159:102154.	Randomized controlled trial/men and women with abdominal obesity without diabetes/2 g fish oil/day (860 mg DHA + 120 mg EPA) (intervention, n = 38) or 2 g corn oil (CO)/day (control, n = 35) for 12 weeks/higher insulin and HOMA-IR at baseline were linked to significant decreases in the fish oil group.	The inflammation of adipose tissue primarily causes insulin resistance (IR). Docosahexaenoic acid (DHA), eicosapentaenoic acid (EPA), and other long-chain omega-3 polyunsaturated fatty acids (LCn-3PUFA) are anti-inflammatory bioactive lipids that may delay the onset of type 2 diabetes (T2D).	Fish oil substantially decreased HOMA-IR by −0.40 units and fasting insulin by −1.62 IU/L when compared to corn oil. At baseline, higher levels of insulin and HOMA-IR were linked to more considerable decreases in the fish oil group. There was no connection between sex and the medication that caused insulin levels to change.	In individuals with abdominal obesity, DHA-enriched fish oil decreases IR; sex-dependent variations were not observed in this investigation.
Zheng et al. [82]	*EBioMedicine*. 2018; 31:150–156.	Randomized controlled trial/150 humans with T2DM/for 180 days, participants in each group received 4 capsules each day, which equaled 2 g of C20:5n-3 and C22:6n-3 in the case of fish oil and 2.5 g of C18:3n-3 in the case of flaxseed oil. Corn oil served as the control oil/omega-3 supplementation has an impact on blood lipid profiles differently in T2D patients with various genotypes at CD36, NOS3, and PPARG.	How genetic variations affect omega-3 fatty acid supplements affect blood lipids is unknown.	The lipid profiles of T2D patients with the CD36-G, PPARG-G, and NOS3-A alleles tended to improve more quickly after treatment with omega-3 fatty acids. The omega-3 fatty acid group’s interaction outcomes were primarily related to fish oil supplementation.	According to this study, omega-3 supplements had a distinct effect on blood lipid profiles in T2D patients with diverse genotypes at CD36, NOS3, and PPARG.
Thota et al. [83]	*Sci Rep*. 2018;8(1):13679.	Randomized, placebo-controlled, and crossover study/human subject/on four test days separated by a week, participants took a placebo, 180 mg of curcumin in the form of tablets, 1.2 g of long-chain omega-3 PUFA in the form of capsules, and 180 mg of fish oil plus curcumin/curcumin reduces postprandial glycemic response and insulin demand for glucose control.	In preclinical investigations, curcumin, a dietary bio-active produced from turmeric and fish oil, reduced fasting blood sugar and insulin resistance.	The curcumin and curcumin + fish oil groups had significantly lower postprandial glucose concentrations.	The postprandial glycemic response and insulin demand for glucose control are decreased by curcumin but not by fish oil.
Makrides et al. [84]	*N Engl J Med*. 2019;381(11):1035–1045.	Randomized controlled trial/human pregnant women/fish oil capsules contained 900 mg of omega-3 PUFA or vegetable oil capsules contained trace omega-3 PUFA (control group) daily, beginning before 20 weeks of gestation and continuing to 34 weeks of gestation or delivery/the omega-3 group reported more minor gastrointestinal issues than the control group did.	N-3 long-chain polyunsaturated fatty acid supplementation during pregnancy may decrease the likelihood of preterm birth and cause the pregnancy to last beyond term.	In the n-3 group, early premature delivery occurred in 61 of 2734 pregnancies and 55 of 2752 pregnancies.	N-3 long-chain polyunsaturated fatty acid supplementation from the beginning of pregnancy to 34 weeks of gestation did not lead to a greater incidence of interventions in post-term deliveries or a lower incidence of early preterm deliveries than control.
Wang et al. [85]	Zhonghua Yu Fang Yi Xue Za Zhi. 2019;53(6):570–575.	Randomized controlled trial/human subjects/3 g/day fish oil (FO), perilla oil (PO), or fish oil mixed with linseed oil (FLO) for 6 months/omega-3 PUFA from various sources had similar impacts on glucose metabolism in type 2 diabetics with dyslipidemia.	Impact of different sources of omega-3 polyunsaturated fatty acids on the glucolipid metabolism in patients with dyslipidemia who have type 2 diabetes.	After 6 months, there were no appreciable differences in serum glucose levels, glycated hemoglobin, C-peptide, insulin, or homeostasis model assessment-insulin resistance between the fish oil, perilla oil, or fish oil combined with linseed oil groups.	Omega-3 PUFA from various sources had comparable effects on glucose metabolism in type 2 diabetics with dyslipidemia. Each of these has a promising future for use in enhancing lipid metabolism.
Talari et al. [86]	*Br J Nutr*. 2019;122(4):423–430.	Randomized controlled trial/vitamin D deficient diabetic human subjects/50,000 IU vitamin D supplements every 2 weeks plus 2× 1000 mg/d n-3 fatty acids from flaxseed oil (n = 30) or placebo (n = 31) for 6 months/co-supplementation of vitamin D and omega-3 fatty acids improved markers of cardiometabolic risk.	This study looked at the impact of co-supplementing with vitamin D and n-3 fatty acids on cardiometabolic risk markers in diabetic patients with CHD.	Compared to the placebo, the co-supplementation of vitamin D and n-3 fatty acids significantly decreased the mean and maximum levels of left carotid intima-media thickness (CIMT) and the mean and maximum levels of right CIMT.	The co-supplementation of n-3 fatty acids and vitamin D reduced cardiometabolic risk markers.
Wasserfurth et al. [87]	*Nutrients*. 2020;12(7):2139.	Randomized controlled trial/healthy human subjects/there were four study groups: (1) a control group receiving no intervention; (2) aerobic and resistance training only for two weeks; (3) exercise combined with dietary advice following the recommendations of the German Nutrition Society; and (4) exercise combined with the consumption of 2 g of *Calanus finmarchicus* oil daily/in senior untrained overweight people, a combination of moderate activity, consumption of *Calanus finmarchicus* oil, and a good diet may improve fat loss.	Harmful changes brought on by aging, such as the emergence of diseases such as type-2 diabetes mellitus or persistent low-grade inflammation, include a steady loss of muscle mass and an increase in fat mass.	The exercise regimen combined with an intake of 2 g/day of oil from the Calanus finmarchicus group and the exercise regimen combined with dietary counseling following the recommendations of the German Nutrition Society group showed the most significant reductions in body fat. Blood lipids and glucose metabolism indicators remained constant across all groups.	In untrained, overweight seniors, moderate activity; consumption of *Calanus finmarchicus* oil; or a good diet may improve fat loss.
Ruan et al. [88]	*Food Funct*. 2019;10(5):2471–2479.	Randomized controlled trial/human subjects with T2DM/fish oil (FO, 4 capsules per day, 50% of EPA + DHA), flaxseed oil (FSO, 4 capsules per day, 63% of ALA), and corn oil (CO, 4 capsules per day, serving as a control) for 180 days/as a potent biomarker of fish oil, 3-carboxy-4-methyl-5-propyl-2-furanpropanoic acid suggested that marine omega-3 PUFA intake may have a positive impact on lipid metabolism and renal function in T2D patients.	The impact of n-3 fatty acid supplements on urine metabolite profile and how they relate to metabolic risk factors in individuals with type 2 diabetes in China.	Compared to the corn oil group, levels of 2-hexenoylcarnitine and 3-carboxy-4-methyl-5-propyl-2-furan propanoic acid (CMPF) were significantly higher in the fish oil group, whereas those of hydroxyisovaleroyl carnitine were much lower.	Marine omega-3 PUFA intake may positively impact lipid metabolism and kidney health in T2D individuals.

## Data Availability

This is a review paper. Data were utilized from open-access resources.
